# Robust and Efficient Dual-Strategy Switch Migration for Failure Recovery in Software-Defined Satellite Networks

**DOI:** 10.3390/s26165163

**Published:** 2026-08-14

**Authors:** Shuang Xu, Zhenyu Yin, Min Huang, Liubin Xing

**Affiliations:** 1 Shenyang Institute of Computing Technology Co., Ltd., Chinese Academy of Sciences, Shenyang 110168, China; 2 College of Artificial Intelligence, Taiyuan University of Technology, Jinzhong 030600, China; 3 College of Information Science and Engineering, Northeastern University, Shenyang 110004, China

**Keywords:** software-defined satellite network, failure recovery, switch migration, adaptive strategy

## Abstract

Software-defined satellite networks (SDSNs) enhance resource utilization and flexibility in space-based networks by leveraging a global view and programmability. A highly reliable control plane is essential to sustain network operations. However, the highly dynamic topology and physical failures in Low Earth Orbit (LEO) environments can cause satellite node outages or inter-satellite link disruptions, leading to control plane interruptions and local load imbalances. To address this, we propose a switch migration mechanism for failure recovery and establish a multi-objective migration model that jointly optimizes control link delay, controller load variance, and normalized migration ratio. To accommodate distinct dynamic characteristics such as frequent topology changes, failure-intensive periods, and stable periods, we design two algorithms: a robust migration algorithm, DNSGA-II, which features population diversity maintenance and environmental awareness, and an efficient migration algorithm, IHAOAVOA, which integrates strong global exploration with powerful local exploitation. Simulation results show that IHAOAVOA rapidly converges under large-scale failures, achieving millisecond-level delay recovery and low normalized migration ratio overhead during failure-intensive periods, while DNSGA-II focuses on long-term load balancing and system stability during stable periods, effectively suppressing localized controller overload. By adopting IHAOAVOA during topology fluctuations or high-failure phases to reduce delay, and switching to DNSGA-II during stable phases to optimize load distribution, the overall network robustness can be improved under the evaluated failure scenarios. This work provides effective support for achieving highly reliable control in SDSNs under failure scenarios.

## 1. Introduction

Withthe rapid development of Low Earth Orbit (LEO) satellite constellations, satellite Internet is increasingly becoming a vital component of the global communication infrastructure. However, the highly dynamic topology, inherent closedness, and limited programmability of satellite networks make it difficult to meet the flexible scheduling and high-reliability demands of future diversified services. To address these issues, the Software-Defined Networking (SDN) paradigm has been introduced into satellite networks, giving rise to software-defined satellite networks (SDSNs), which decouple the control plane from the data plane to enable centralized control, unified management, and on-demand reconfiguration. In an SDSN architecture, controllers are responsible for collecting status information from satellite switches, computing optimal routes, and distributing forwarding rules; the locations where controllers are deployed and the quality of control paths directly determine the overall service performance of the network [1]. In practical operation, however, the high-speed movement of LEO satellites leads to frequent periodic topology changes, readily causing inter-satellite link (ISL) disruptions and failures. Meanwhile, the harsh space environment often renders satellite nodes incapable of communication due to factors such as spatial obstructions, energy shortage, or hardware failures [2]. Once such abnormal events occur, the original control paths between controllers and switches can be damaged, increasing the risk of instantaneous communication interruptions. In these failure scenarios, failure to promptly adjust the mapping between switches and controllers will significantly increase control delay, aggravate controller load imbalance, and incur high network reconfiguration overhead, ultimately jeopardizing the stable operation of the control plane. Therefore, establishing an efficient and reliable failure recovery and switch migration mechanism has become essential to ensuring the stable operation and service quality of LEO satellite networks.

Existing studies have primarily focused on the dynamic topology characteristics of SDSNs and have proposed various optimization models for controller deployment [3] and switch-to-controller mapping [4], thereby ensuring control efficiency and load balancing to a certain extent under ideal operating conditions [5]. Nevertheless, these pre-planned provisioning schemes largely rely on predictable orbital regularities and are ill-suited to cope with sudden link failures and node outages caused by harsh space environments or equipment anomalies. In such failure scenarios, established control associations are subject to instantaneous disruptions. Conventional static or quasi-static recovery strategies, lacking awareness of multi-objective conflicts and the capability for adaptive fine-tuning, typically result in elevated control delays, excessive network reconfiguration overhead, and even secondary controller overload. Therefore, to handle abrupt topology changes induced by unforeseen failures, it is imperative to devise an adaptive switch migration mechanism that reconciles high timeliness with strong robustness, so as to preserve the continuity and global stability of the control plane. Recent studies on inter-satellite networking have jointly considered controller placement and switch assignment, while research on control-plane fault tolerance has increasingly investigated redundant controller placement and proactive recovery. However, these studies generally do not explicitly characterize the joint trade-offs among post-failure control-path delay, controller load variance, and the normalized migration ratio.

Focusing on the susceptibility of control paths to disruption and the sudden surge in controller load under link failures or node outages in SDSNs [6], this paper investigates a robust and efficient dual-strategy switch migration mechanism for failure recovery. Given that control associations must be rapidly adjusted under failure conditions while multiple performance metrics are mutually constrained, we formulate a multi-objective switch migration optimization model with the objectives of minimizing the average control-path delay, controller load variance, and normalized migration ratio. Furthermore, considering the large search space and complex coupling among decision variables inherent in failure recovery, we design two categories of intelligent optimization-based migration strategies: a robust multi-objective migration strategy based on the Dynamic Non-dominated Sorting Genetic Algorithm II (DNSGA-II), and an efficient search strategy based on the Improved Hybrid Aquila Optimizer–African Vultures Optimization Algorithm (IHAOAVOA), catering to stability-prioritized and rapid recovery requirements, respectively. The main contributions of this paper are as follows:

- A failure-recovery-oriented switch migration model is established for software-defined satellite networks. The model explicitly considers link failures, controller node failures, and switch node failures, and jointly evaluates control-path delay, controller load variance, and the normalized migration ratio.
- A DNSGA-II-based migration strategy is developed for scenarios in which controller load stability is the primary concern. By reusing elite solutions from the previous network snapshot and combining non-dominated sorting with crowding-distance-based selection, the method improves solution continuity under time-varying network conditions.
- Composite opposition-based learning (COBL), fitness–distance balance (FDB), and discrete search mechanisms are integrated into the IHAOAVOA-based migration strategy to enhance global exploration and local exploitation in failure-prone and highly dynamic scenarios. The main contribution lies in adapting and combining these mechanisms under the discrete mapping constraints of SDSNs, rather than redefining the underlying optimization operators.

The remainder of this paper is organized as follows. Section 2 reviews related work. Section 3 establishes the system model and formulates the problem. Section 4 elaborates on the two switch migration mechanisms. Section 5 presents the simulation results and performance analysis. Section 6 concludes the paper.

## 2. Related Works

Existing studies can be broadly organized into three research streams. The first exploits orbital predictability to jointly optimize controller placement and switch assignment. The second adjusts control domains across time slots or topology snapshots to accommodate periodic topology changes. The third addresses control-plane failures through redundant controller placement, proactive fault tolerance, or route reconstruction. The following subsections review these studies from the perspectives of dynamic controller deployment, failure recovery, and intelligent optimization.

### 2.1. Dynamic Controller Deployment in Software-Defined Satellite Networks

To cope with the highly dynamic and periodically time-varying topology of LEO satellite networks, existing studies predominantly exploit the predictability of orbital motion, giving rise to two main technical approaches: ephemeris-based pre-planning and time-slot- or snapshot-based dynamic migration. One line of work focuses on the advance planning of controller locations based on orbital ephemeris. Li et al. [7] proposed an optimized controller provisioning strategy that adaptively adjusts both the number and positions of satellite controllers by combining short-term traffic prediction with long-term orbital ephemeris prediction, aiming to minimize the total control overhead throughout the satellite operational period. However, the optimization underlying this strategy rests on the assumption that the network operates normally according to the precomputed ephemeris, leaving no margin for random node failures or transient link occlusions. Once an unforeseen physical contingency occurs, the pre-planned deployment becomes mismatched with the actual topology. Guo et al. [8] leveraged the predictability of satellite orbits and proposed an optimization scheme that integrates static placement with dynamic assignment. By precomputing satellite visibility windows, control associations are reassigned prior to significant topology changes, thereby reducing handover frequency and control delay. This work likewise treats all topological evolution as a consequence of deterministic orbital mechanics, lacking any response mechanism for sudden control-node outages caused by high-energy particles in space. Once the master controller fails, the switches under its jurisdiction remain uncontrolled until the next reassignment cycle. Jiang et al. [9] constructed an ephemeris-based spatiotemporal topology model to jointly optimize controller placement and switch assignment, mitigating the signaling storms induced by frequent topology changes through precomputation of optimal control domain partitioning at multiple time points. These ephemeris-prediction-dependent planning methods equate network dynamics entirely with predictable periodic variations, ignoring the numerous unpredictable sudden failures in the space environment. When transient local link disconnections occur, the control plane, lacking adaptive reconfiguration capability, will suffer large-scale interruptions.

To overcome the limitations of purely prediction-based approaches and to accommodate actual topology evolution, another mainstream class of solutions partitions the continuous dynamic process into a series of discrete time slots or snapshots and dynamically migrates control ownership across different time intervals. Wang et al. [10] designed a two-layer software-defined architecture for mega-constellations, dividing the network into multiple time slots corresponding to stable topology snapshots and migrating control authority at slot boundaries according to a predefined policy to take over network management tasks. While this mechanism improves control-plane survivability to some extent, it lacks the capability for local adaptive fine-tuning in response to sudden failures. When a local node experiences instantaneous failure, it can easily trigger a cascade of blind migrations among numerous healthy nodes, thereby incurring substantial network reconfiguration overhead. Peng et al. [11] proposed a collaborative control plane architecture that employs a time-slot mechanism to dynamically adjust the control scope of satellite group controllers and migrates control ownership at slot boundaries to reduce end-to-end delay. However, this method relies solely on large-scale mapping adjustments across time slots; when a local control link is abnormally disrupted, a large number of affected switches instantaneously issue takeover requests to neighboring healthy controllers, which can readily cause severe secondary local overload. Furthermore, Tang et al. [12] proposed a reinforcement-learning-based control domain partitioning method that dynamically learns the optimal control ownership migration strategy within each discrete time slot for the current snapshot. This framework likewise suffers from a coarse temporal response granularity, failing to precisely constrain the burst traffic that floods in within a short period during instantaneous failures, leading to network oscillations during the transition phase between snapshots. Existing discretized migration schemes, when confronted with emergency failure-recovery scenarios characterized by conflicting multiple objectives, still struggle to simultaneously achieve timely failure recovery and global control robustness.

### 2.2. Fault Tolerance and Migration Mechanisms for Failure Recovery

In software-defined satellite networks, where sudden node outages occur frequently due to harsh space environments, fault tolerance and rapid takeover mechanisms in the control plane are critical to ensuring high network availability. Chang et al. [13] developed a failure-aware optimization model for network failure scenarios that predetermines optimal redundant positions for controllers, so that backup nodes can take over network management tasks with minimal delay. However, such precomputed static redundancy schemes primarily focus on network-wide deployment optimization and overlook the multidimensional trade-offs among control-path delay, local controller load, and the normalized migration ratio when numerous disconnected switches must dynamically connect to backup controllers immediately after a failure. Furthermore, Deng et al. [14] proposed a dynamic switch migration strategy that combines deep reinforcement learning with heuristic algorithms, proactively migrating some switches before the primary controller fails to achieve failure prevention. Despite the use of intelligent optimization techniques, reinforcement-learning models trained through interactions with historical network states may suffer from state-distribution mismatch under large-scale and unpredictable changes in the space environment. Moreover, their accompanying heuristic rules may struggle to identify, within a limited recovery window, a globally competitive solution that simultaneously minimizes the normalized migration ratio and post-reassignment controller load variance. These studies indicate that purely predefined fault tolerance and experiential learning cannot fully adapt to extreme burst environments, and the affected disconnected switches require a dynamic migration mechanism capable of adaptive multi-objective trade-offs after a failure.

To address the loss of connectivity caused by complete satellite link failures or physical damage to nodes, existing studies have primarily focused on restoring communication connectivity through route reconfiguration and switch remapping. Du et al. [15] proposed a fast rerouting mechanism based on segment routing and dual-timer switchover, utilizing pre-installed backup paths in the data plane to achieve millisecond-level traffic redirection upon link failure. However, this mechanism is largely confined to local detours in the data plane and does not involve the reassignment of switch ownership in the control plane. When the control link itself suffers a physical disruption, this strategy cannot resolve the problem of orphaned switches actively migrating to a new controller.

Jamal et al. [16] introduced graph neural networks (GNNs) to model the complex topology of satellite constellations and dynamically predict link states to generate optimal routing strategies for proactive route reconfiguration. Yet such prediction-based reconfiguration heavily depends on the evolutionary regularities of historical topology. When confronted with unpredictable physical destruction, such as a sudden high-energy particle strike, the prediction model instantly becomes invalid. If it degrades into reactive emergency remapping, a greedy nearest-access principle is often adopted, causing the controller closest to the failure point to experience severe secondary load imbalance due to a sudden influx of takeover requests. Furthermore, Wang et al. [17] designed a proactive fault-tolerant approach based on traffic engineering, which prevents cascading failures in node outage scenarios by precomputing backup paths and dynamically adjusting traffic distribution. However, in the context of control domain reconfiguration with conflicting multiple objectives, pure traffic steering strategies are difficult to contain the overhead of large-scale switch remapping, and blind remapping without global constraints can induce substantial unnecessary local oscillations in the network, resulting in high reconfiguration costs. Existing failure-recovery mechanisms remain constrained by greedy, single-objective strategies when responding to unexpected disconnections. Therefore, developing an adaptive migration model that jointly achieves low control-path delay, balanced controller loads, and a low normalized migration ratio remains an important open challenge.

### 2.3. Application of Intelligent Optimization Algorithms in Network Reconfiguration

Once the multi-objective switch migration model is established, the mapping reconfiguration process is essentially a highly complex NP-hard combinatorial optimization problem, making it intractable for conventional exact algorithms to solve within polynomial time. Consequently, metaheuristic algorithms have become the mainstream approach in this research domain. Zhao et al. [18] addressed the scenario of random inter-satellite link failures in LEO constellations by constructing a constrained model incorporating task revenue, handover frequency, and routing cost, and explored feasible paths under multiple constraints. However, this method relies on a discrete-time strategy and a static topology graph; when coping with highly dynamic topological changes induced by physical failures, it typically incurs substantial computational overhead to maintain solution validity, thus failing to meet the instantaneous recovery requirements within a short time window. Li et al. [19] introduced an ant colony algorithm with delay and available wavelength ratio as heuristic functions in SDSN-based satellite optical networks, aiming to optimize cross-layer wavelength routing to reduce network congestion. Nevertheless, such optimization methods based on static or quasi-static snapshots lack the capability of deep memory for temporal topology evolution. When a sudden failure occurs, the algorithm cannot reuse historical structural information and must be re-initialized, undergoing numerous redundant iterations, thereby significantly increasing the recovery delay. Qi et al. [20] constructed a topology reconstruction strategy for satellite-terrestrial integrated networks to address the vulnerability and high mobility of satellite nodes. Traditional memoryless optimization methods impose a significant computational burden in extreme environments characterized by resource shortages and drastic topology changes, making it difficult to strike an ideal balance between real-time computational efficiency and global reconfiguration performance. At the level of routing and topology reconfiguration, conventional metaheuristic algorithms suffer from an inherent contradiction between convergence speed and solution quality due to the lack of a warm-start mechanism based on historical states, rendering them ill-suited to the stringent time constraints at the moment a failure occurs.

In terms of controller deployment, load balancing, and joint resource optimization in SDSNs, intelligent algorithms likewise confront severe challenges posed by high-dimensional discrete mapping. Pan et al. [21] employed an improved artificial bee colony algorithm for topology reconstruction in space information networks, enhancing network survivability by comprehensively considering satellite node visibility and connectivity duration. However, when applied to large-scale LEO constellations, the solution space of the discrete mapping grows exponentially, which tends to induce premature convergence in basic swarm intelligence algorithms, thereby trapping them in local optima and preventing them from discovering the globally optimal architecture. Sun et al. [22] attempted to use genetic and particle swarm algorithms to address strongly time-correlated resource competition constraints in satellite IoT service deployment strategies. Yet such algorithms typically enforce an artificial discretization of time slots, severing the continuous evolution pattern of link states, which easily leads to state discontinuities or infeasibility in the generated scheduling solutions at temporal boundaries. As noted by Dong et al. [23] in their research on delay-sensitive services, conventional metaheuristic-based stochastic search methods struggle to maintain the long-term stability of the control architecture while guaranteeing low-latency response. In summary, existing metaheuristic algorithms are essentially memoryless stochastic search strategies that lack the capability to model topological evolution regularities and perform adaptive optimization. It is therefore necessary to introduce novel intelligent algorithms equipped with historical temporal memory and multi-state adaptive coordination, in order to resolve the challenging problem of achieving both timely and highly stable reconfiguration in SDSNs under multiple concurrent failures. To overcome the limitations of conventional heuristic algorithms in handling the highly dynamic and high-dimensional failure solution space of SDSNs, this paper proposes two tailored improved algorithms that incorporate the physical and resource characteristics of satellite networks. On the one hand, we propose the DNSGA-II algorithm, which accelerates post-failure convergence by introducing a dynamic population prior knowledge retention mechanism that inherits information from the previous time slot, thereby satisfying scenarios with stringent long-term load stability requirements. On the other hand, we design an Improved Hybrid Aquila Optimizer–African Vultures Optimization Algorithm (IHAOAVOA), which enhances population diversity through a composite opposition-based learning and fitness–distance balance mechanism, effectively mitigating premature convergence and enabling the efficient derivation of delay-optimal switch-to-controller mappings immediately after a failure.

Beyond controller placement and switch assignment, recent research on satellite–terrestrial integrated networks has jointly considered service deployment, task offloading, routing, and topology reconstruction. These studies demonstrate the strong coupling between network-function placement and routing decisions in highly dynamic satellite environments, particularly when computing resources, link availability, and delay-sensitive services must be considered simultaneously [20,22,23]. Nevertheless, their primary focus is service provisioning or data-plane routing during normal network evolution, rather than the reactive reconstruction of switch-to-controller associations following unexpected failures.

Control-plane fault-tolerance studies have investigated redundant controller placement, proactive failure avoidance, backup-path planning, and learning-based switch migration [10,13,14,17]. Although these approaches improve network survivability, they generally emphasize pre-planned redundancy, failure prevention, or a single recovery objective. The joint optimization of post-failure control-path delay, controller load variance, and the normalized migration ratio during reactive switch reassignment has received limited attention. To address this gap, this study formulates switch-to-controller reassignment after link or node failures as a multi-objective recovery problem and investigates two complementary strategies that prioritize recovery efficiency and control-plane stability, respectively.

## 3. System Model and Problem Formulation

The design of a failure-recovery-oriented switch migration mechanism requires a well-defined network structure representation, a method for measuring control path performance, and a controller load model, upon which the optimization problem can be subsequently formulated. This section presents the system model of the SDSN, encompassing the dynamic satellite network topology model, the control path delay model, and the load modeling approach, and then defines the multi-objective switch migration optimization problem in failure-recovery scenarios.

### 3.1. Dynamic Topology and Failure Model

The physical topology of an SDSN exhibits highly dynamic and time-varying characteristics due to the high-speed orbital motion of satellites. To capture this dynamic evolution process, we model the satellite network topology as a time-varying graph (TVG) G=(V,E,T,ϕ), where *V* denotes the set of all satellite nodes deployed in the network. The node set can be further partitioned into a set of switch nodes $S=\{s_{1},s_{2},\ldots ,s_{M}\}$ and a set of controller nodes $C=\{c_{1},c_{2},\ldots ,c_{K}\}$ satisfying V=S∪C.E⊆V×V represents the set of potential inter-satellite links. Considering the periodicity and trajectory predictability of LEO satellite motion, we adopt a time-slot snapshot mechanism that divides the continuous system operation period into a sequence of discrete, equal-length micro time slots $T=\{t_{1},t_{2},\ldots ,t_{N}\}$. In satellite networks, whether a link can be established depends first on whether the distance between two satellite nodes satisfies the communication requirement; a link between two satellite nodes *u* and *v* must meet the maximum communication distance constraint. Let the position vector of satellite *u* in the inertial coordinate system be $r_{u}\left(t_{k}\right)$ and that of satellite *v* be $r_{v}\left(t_{k}\right)$. Then, the distance $\rho_{uv}$ between them can be computed via the Euclidean norm as follows:

(1) $$\rho_{uv}={∥r_{u}\left(t_{k}\right)-r_{v}\left(t_{k}\right)∥}_{2}$$

This distance must satisfy the necessary condition for link existence, $\rho_{uv}\leq \rho_{\max}$, where $\rho_{\max}$ is determined by the onboard antenna’s transmit power and receiving sensitivity. This condition ensures that the signal strength during propagation is sufficient to be captured by the receiving end.

In addition to the communication-distance constraint, an inter-satellite link must satisfy the Earth-blockage constraint. Let $r_{u}\left(t_{k}\right)$ and $r_{v}\left(t_{k}\right)$ denote the position vectors of satellites *u* and *v*, respectively, at time slot $t_{k}$. The geocentric angle between the two satellites is defined as follows:

(2) $$\theta_{uv}\left(t_{k}\right)=\arccos\left(\frac{r_{u}^{T}\left(t_{k}\right)r_{v}\left(t_{k}\right)}{{∥r_{u}\left(t_{k}\right)∥}_{2}{∥r_{v}\left(t_{k}\right)∥}_{2}}\right).$$

The inter-satellite line of sight is not blocked by the Earth if

(3) $$\theta_{uv}\left(t_{k}\right)\leq \arccos\left(\frac{R_{e}+h_{\mathrm{safe}}}{{∥r_{u}\left(t_{k}\right)∥}_{2}}\right)+\arccos\left(\frac{R_{e}+h_{\mathrm{safe}}}{{∥r_{v}\left(t_{k}\right)∥}_{2}}\right).$$

When condition (3) is satisfied, the line segment connecting the two satellites does not intersect the safety sphere of radius $R_{e}+h_{\mathrm{safe}}$ centered at the Earth. Therefore, the inter-satellite line of sight is considered unobstructed by the Earth. Here, $R_{e}$ denotes the Earth’s radius, and $h_{\mathrm{safe}}$ is a height margin introduced to account for orbital uncertainties and provide a conservative clearance. The link-state function jointly evaluates the communication-distance threshold and the above line-of-sight condition.

We further define the on/off status of a link. Since the static modeling of satellite network topology is typically performed within discrete time slots, a function capable of evaluating the link status in each slot is required. Let ϕ:E×T→{0,1} be the edge existence function, satisfying $\phi \left(e,t_{k}\right)=e_{uv}\left(t_{k}\right)$.The edge existence function $\phi (e,t_{k})$ explicitly indicates whether an arbitrary link is connected in a specific time slot:

(4) $$\phi \left(e,t_{k}\right)=\left\{\begin{array}{cc} 1, & \forall t\in \left[t_{k},t_{k}+\Delta t\right],\;\rho_{uv}\left(t_{k}\right)\leq \rho_{\max}\;\mathrm{and}\;\theta_{uv}\left(t_{k}\right)\leq \theta_{\max}\left(t_{k}\right)\\ 0, & \mathrm{otherwise} \end{array}\right.$$

where for any two satellite nodes *u* and *v* in the network, $\rho_{uv}$ denotes the Euclidean distance between *u* and *v* and this distance must not exceed the maximum communication distance threshold $\rho_{\max}$

The matrix $A^{k}={\left(a_{ij}\right)}_{n\times n}$ represents the assignment relationship between switches and controllers in time slot $t_{k}$:

(5) $$a_{ij}=\left\{\begin{array}{cc} 1, & \mathrm{if}\;\mathrm{switch}\;s_{j}\;\mathrm{is}\;\mathrm{assigned}\;\mathrm{to}\;\mathrm{controller}\;c_{i}\\ 0, & \mathrm{otherwise} \end{array}\right.$$

where $a_{ij}$ indicates whether switch $s_{j}$ belongs to controller $c_{i}$. Specifically, $a_{ij}\left(t_{k}\right)=1$ if and only if switch $s_{j}\in S$ is successfully assigned to the control domain of controller $c_{i}\in C$. When the control authority of a switch migrates between controllers, it causes a change in the assignment matrix,

(6) $$\left\{\begin{array}{c} a_{ij}\leftarrow 0\\ a_{\xi j}\leftarrow 1 \end{array}\right.$$

which means that the control authority of switch $s_{j}$ migrates from controller $c_{i}$ to $c_{\xi }$. To capture the sudden component failures occurring during SDSN operation, we construct a failure state mutation model based on the aforementioned graph model, focusing on characterizing the disruptive impact of both link failures and node failures on the network topology and control logic. First, when a link failure occurs, the connectivity state function of the affected inter-satellite link e=(u,v) abruptly changes to 0:

(7) $$\phi \left(e,t_{k}\right)=0,\;e=\left(u,v\right)\in E$$

This localized loss of topological connectivity triggers network-wide route reconfiguration, directly altering the shortest-path distance matrix between nodes and thereby increasing the communication delay between the control plane and the data plane. Second, when a node failure occurs, a certain satellite node completely loses its communication capability, causing all inter-satellite links incident to it to disconnect simultaneously. Suppose node *u* fails within time slot $t_{k}$; then the existence function of all inter-satellite link edges $e_{uv}$ incident to node *u* in that time slot becomes 0, i.e.,

(8) $$\phi \left(e,t_{k}\right)=0,\;\forall e=\left(u,v\right)\in E$$

Let *C* denote the set of all controllers in the network. If controller node $c_{f}$ fails within time slot $t_{k}$, the set of available controllers immediately becomes $C^{\prime }=C\setminus \left\{c_{f}\right\}$. Let $S_{c_{f}}\left(t_{k}\right)$ be the set of switch nodes originally belonging to the failed controller $c_{f}$. All switch nodes in this set must then migrate and re-assign to other healthy controllers $c_{\xi }$. Accordingly, the elements of the updated assignment matrix $A^{k}$ change as follows:

(9) $$a_{c_{f},j}\left(t_{k}\right)\leftarrow 0,\;a_{c_{\xi },j}\left(t_{k}\right)\leftarrow 1,\;c_{\xi }\in C^{\prime },\forall s_{j}\in S_{c_{f}}\left(t_{k}\right)$$

In time slot $t_{k}$, the failure-recovery delay $D_{\mathrm{controller}\_\mathrm{fail}}\left(t_{k}\right)$ and the failure-recovery load $L_{\mathrm{controller}\_\mathrm{fail}}\left(t_{k}\right)$ caused by the controller node failure are respectively expressed as

(10) $$D_{\mathrm{controller}\_\mathrm{fail}}\left(t_{k}\right)=\sum_{j\in S_{c_{f}}\left(t\right)}d_{j,c_{\xi }}\left(t_{k}\right)$$

(11) $$L_{\mathrm{controller}\_\mathrm{fail}}\left(t_{k}\right)=\sum_{j\in S_{c_{f}}\left(t\right)}L_{j}\left(t_{k}\right)$$

where $d_{j,c_{\xi }}\left(t_{k}\right)$ denotes the delay from switch $s_{j}$ to the newly selected controller $c_{\xi }$, and $L_{j}\left(t_{k}\right)$ denotes the load of switch $s_{j}$ in time slot $t_{k}$.

When switch node $s_{f}$ fails within time slot $t_{k}$ where $s_{f}$ is originally controlled by controller $c_{i}$, the control association between this node and the controller is severed, which can be expressed as

(12) $$a_{i,s_{f}}\left(t_{k}\right)=0,\;\forall s_{f}\in S$$

After switch node $s_{f}$ fails, the traffic load $L_{s_{f}}\left(t_{k}\right)$ previously handled by this node is redistributed among the available neighboring switches in $S_{\mathrm{neighbor}}\left(s_{f}\right)$. Let $\beta_{f,s^{\prime }}\left(t_{k}\right)$ denote the proportion of the failed switch’s traffic load assigned to neighboring switch $s^{\prime }$. The load of each neighboring switch is then updated as follows:

(13) $$L_{s^{\prime }}\left(t_{k}\right)\leftarrow L_{s^{\prime }}\left(t_{k}\right)+\beta_{f,s^{\prime }}\left(t_{k}\right)L_{s_{f}}\left(t_{k}\right),\;s^{\prime }\in S_{\mathrm{neighbor}}\left(s_{f}\right),$$

subject to

(14) $$\beta_{f,s^{\prime }}\left(t_{k}\right)\geq 0,\;\sum_{s^{\prime }\in S_{\mathrm{neighbor}}\left(s_{f}\right)}\beta_{f,s^{\prime }}\left(t_{k}\right)=1.$$

Here, $\beta_{f,s^{\prime }}\left(t_{k}\right)$ is determined by the recovery strategy, and the allocation ratios sum to one to ensure that the traffic load of the failed switch is completely redistributed. The set $S_{\mathrm{neighbor}}\left(s_{f}\right)$ contains only neighboring nodes that remain available and satisfy the connectivity requirements after the failure. Based on the updated loads of these neighboring nodes, the recovery load induced by the switch-node failure is defined as follows:

(15) $$L_{\mathrm{switch}\_\mathrm{fail}}\left(t_{k}\right)=\sum_{s^{\prime }\in S_{\mathrm{neighbor}}\left(s_{f}\right)}L_{s^{\prime }}\left(t_{k}\right)$$

### 3.2. Multi-Objective Optimization Model

To reconstruct the control associations after a failure and maintain the long-term operational stability of the network, we formulate a multi-objective optimization model based on the dynamic assignment variable $a_{ij}\left(t_{k}\right)$. The model jointly considers the average control-path delay, controller load variance, and the normalized switch migration ratio.

First, the control path delay directly determines the real-time performance of flow rule delivery and network state collection. After a failure occurs, switches must reselect surviving backup controllers. Let $d_{ij}\left(t_{k}\right)$ denote the path delay between switch $s_{j}\in S^{\prime }$ and controller $c_{i}\in C^{\prime }$ based on the current connectivity graph in time slot $t_{k}$. The network-wide average control-path delay, denoted by $D_{\mathrm{total}}\left(t_{k}\right)$, is defined as follows:

Here, $P_{ij}\left(t_{k}\right)$ denotes an available control path from switch $s_{j}$ to controller $c_{i}$ in the current connected graph at time slot $t_{k}$. In the experimental implementation, the delay values are obtained from a preprocessed normalized delay matrix. The corresponding unnormalized physical quantity accounts only for the propagation delay along the path; node-processing and queuing delays are not modeled separately. Given the physical link lengths, the raw propagation delay is first calculated and then normalized using the same preprocessing procedure adopted for the experimental data:

(16) $${\tilde{d}}_{ij}\left(t_{k}\right)=\sum_{e\in P_{ij}\left(t_{k}\right)}\frac{\ell_{e}\left(t_{k}\right)}{c},\;d_{ij}\left(t_{k}\right)=\mathrm{Norm}\;\left({\tilde{d}}_{ij}\left(t_{k}\right)\right),$$

(17) $$D_{\mathrm{total}}\left(t_{k}\right)=\frac{1}{\left|S^{\prime }\right|}\sum_{c_{i}\in C^{\prime }}\sum_{s_{j}\in S^{\prime }}d_{ij}\left(t_{k}\right)\;a_{ij}\left(t_{k}\right).$$

Here, $d_{ij}\left(t_{k}\right)$ denotes the normalized control-path delay between controller $c_{i}$ and switch $s_{j}$ at time slot $t_{k}$, while $C^{\prime }$ and $S^{\prime }$ denote the sets of currently available controllers and switches, respectively. This metric represents the average control-path delay across all available switches. Minimizing this metric facilitates the timely delivery of control instructions after failure recovery and reduces the risk of prolonged loss of network control.

Failure recovery may cause traffic to be concentrated on a small number of surviving controllers. Therefore, the controller load variance is adopted to quantify the degree of load imbalance. Let $L_{j}\left(t_{k}\right)$ denote the traffic load of switch $s_{j}$ at time slot $t_{k}$. The aggregate load of controller $c_{i}$ is calculated as

$$L_{c_{i}}\left(t_{k}\right)=\sum_{s_{j}\in S^{\prime }}a_{ij}\left(t_{k}\right)L_{j}\left(t_{k}\right),$$

where $S^{\prime }$ denotes the set of currently available switches. The mean load and load variance of the available controllers are defined as

(18) $$\begin{array}{cc} \bar{L}\left(t_{k}\right) & =\frac{1}{K}\sum_{c_{i}\in C^{\prime }}L_{c_{i}}\left(t_{k}\right),\\ \Delta L_{\mathrm{total}}\left(t_{k}\right) & =\frac{1}{K}\sum_{c_{i}\in C^{\prime }}{\left(L_{c_{i}}\left(t_{k}\right)-\bar{L}\left(t_{k}\right)\right)}^{2}, \end{array}$$

where $K=|C^{\prime }|$ is the number of currently available controllers, and $C^{\prime }$ denotes the corresponding controller set. A smaller value of $\Delta L_{\mathrm{total}}\left(t_{k}\right)$ indicates a more balanced distribution of traffic among the available controllers. This variance-based definition is consistent with the controller load-imbalance metric used in the experimental implementation.

Frequent changes in controller associations generate additional signaling overhead and may cause control-plane oscillations. Consistent with the experimental implementation, the migration metric is defined as the normalized migration ratio, namely, the number of non-controller nodes whose controller associations change between two consecutive time slots divided by the total number of network nodes:

(19) $$M\left(t_{k}\right)=\frac{1}{N}\sum_{s_{j}\in S^{\prime }}1\;\left[c_{j}\left(t_{k}\right)\neq c_{j}\left(t_{k-1}\right)\right],$$

where $c_{j}\left(t_{k}\right)$ denotes the controller associated with switch $s_{j}$ at time slot $t_{k}$, *N* is the total number of network nodes, and 1[·] is the indicator function. A smaller value of $M(t_{k})$ indicates fewer controller-association changes during failure recovery and, consequently, a more stable control plane.

Based on the above three performance metrics, the failure-recovery-oriented switch migration problem is formulated subject to assignment uniqueness, path availability, delay, and controller-capacity constraints. For each experimental configuration, the controller locations are predetermined and remain fixed during switch migration. Therefore, controller deployment is not treated as a decision variable. Let $C^{\prime }$ and $S^{\prime }$ denote the sets of available controllers and switches after a failure, respectively. The decision variable is the assignment matrix $A^{k}={\left[a_{ij}\left(t_{k}\right)\right]}_{|C^{\prime }\left|\times \right|S^{\prime }|}$, where $a_{ij}\left(t_{k}\right)=1$ indicates that switch $s_{j}$ is assigned to controller $c_{i}$ at time slot $t_{k}$, and $a_{ij}\left(t_{k}\right)=0$ otherwise. In addition, let $q_{ij}\left(t_{k}\right)\in \left\{0,1\right\}$ denote the path-availability parameter, where $q_{ij}\left(t_{k}\right)=1$ if an available control path exists between $s_{j}$ and $c_{i}$, and $q_{ij}\left(t_{k}\right)=0$ otherwise. The optimization model is expressed as follows:

(20) $$\min F=w_{D}\;\cdot \;D_{\mathrm{total}}\left(t_{k}\right)+w_{L}\;\cdot \;\Delta L_{\mathrm{total}}\left(t_{k}\right)+w_{M}\;\cdot \;M\left(t_{k}\right)$$

(21) $$\begin{array}{cccc} s.t.\;\; & a_{ij}\left(t_{k}\right)\in \left\{0,1\right\},\; & \; & \forall c_{i}\in C^{\prime },\;s_{j}\in S^{\prime },\\ \; & \sum_{c_{i}\in C^{\prime }}a_{ij}\left(t_{k}\right)=1,\; & \; & \forall s_{j}\in S^{\prime },\\ \; & a_{ij}\left(t_{k}\right)\leq q_{ij}\left(t_{k}\right),\; & \; & \forall c_{i}\in C^{\prime },\;s_{j}\in S^{\prime },\\ \; & d_{ij}\left(t_{k}\right)a_{ij}\left(t_{k}\right)\leq \Delta_{\mathrm{th}},\; & \; & \forall c_{i}\in C^{\prime },\;s_{j}\in S^{\prime },\\ \; & \sum_{s_{j}\in S^{\prime }}a_{ij}\left(t_{k}\right)L_{j}\left(t_{k}\right)\leq L_{i}^{\max},\; & \; & \forall c_{i}\in C^{\prime }. \end{array}$$

In the above formulation, $w_{D}$, $w_{L}$, and $w_{M}$ are non-negative weight coefficients representing the preferences for the average control-path delay, controller load variance, and normalized migration ratio, respectively. The first constraint defines the binary switch-to-controller assignment variable. The second constraint ensures that each available switch is assigned to exactly one available controller. The third constraint prohibits a switch from being assigned to a controller when no available control path exists between them. The fourth constraint requires the control-path delay of an active assignment to remain below the preset threshold $\Delta_{\mathrm{th}}$. The fifth constraint ensures that the aggregate traffic load assigned to controller $c_{i}$ does not exceed its processing capacity $L_{i}^{\max}$.

## 4. Switch Migration Optimization Algorithms for Failure Recovery

In failure scenarios, the topology and link reachability of satellite networks undergo drastic changes, causing the original control paths of some switches to fail or deteriorate in performance. To rapidly restore control plane stability in such dynamic environments, it is essential to design a switch migration mechanism capable of jointly optimizing the average control-path delay, controller load variance, and normalized migration ratio. Based on the multi-objective optimization model, this chapter proposes two categories of migration algorithms from distinct perspectives: a migration strategy based on the Dynamic Non-dominated Sorting Genetic Algorithm II (DNSGA-II), and a migration strategy based on the Improved Hybrid Aquila Optimizer–African Vultures Optimization Algorithm (IHAOAVOA). These two algorithms are respectively suited for load-balancing-sensitive scenarios and highly dynamic search scenarios.

### 4.1. DNSGA-II-Based Switch Migration Mechanism

For mission scenarios demanding high stability and sensitivity to controller load fluctuations, this paper adopts an optimization approach based on the DNSGA-II. Unlike the conventional NSGA-II, DNSGA-II retains the optimal solutions from the previous time slot $t_{k-1}$ and incorporates them as a significant component of the initial population at the current time slot $t_{k}$, thereby improving convergence speed and solution continuity under dynamic topology changes. Its core mechanisms are described as follows:

To ensure that the algorithm preferentially retains migration solutions that perform well across all metrics, in each iteration, all candidate solutions are first ranked comprehensively according to the total control path delay $D_{\mathrm{total}}\left(t_{k}\right)$, the controller load variance $\Delta L_{\mathrm{total}}\left(t_{k}\right)$, and the normalized migration ratio $M(t_{k})$. Each individual $X_{i}$ represents a switch-to-controller mapping scheme within the current time slot. The objective function vector of an individual is given by

(22) $$F\left(X_{i}\right)=\left(D_{\mathrm{total}}^{\left(i\right)}\left(t_{k}\right),\;\Delta L_{\mathrm{total}}^{\left(i\right)}\left(t_{k}\right),\;M^{\left(i\right)}\left(t_{k}\right)\right)$$

To avoid converging to unreasonable solutions when simultaneously optimizing conflicting objectives, the following update rule is established: an individual $X_{a}$ is replaced by another individual $X_{b}$ only if $X_{b}$ is strictly better in at least one objective and no worse in all other objectives.

To avoid convergence to locally concentrated regions, the “degree of isolation” of each individual in the objective space is further computed for the sorted candidate set. For any sorted solution $X_{i}$, let its neighbor distance tothe *k*-th objective function be denoted as

(23) $$dis_{i}^{\left(k\right)}=\frac{f_{k}\left(X_{i+1}\right)-f_{k}\left(X_{i-1}\right)}{f_{k}^{\max}-f_{k}^{\min}}$$

The total crowding distance of this solution is then

(24) $$CD\left(X_{i}\right)=\sum_{k=1}^{3}dis_{i}^{\left(k\right)}$$

where $f_{k}\in \left\{D_{\mathrm{total}},\Delta L_{\mathrm{total}},M\right\}$, and $f_{k}^{\max}$ and $f_{k}^{\min}$ denote the maximum and minimum values of the *k*-th objective in the current population, respectively. A larger crowding distance indicates that the solution is less clustered in the multi-objective space, thereby favoring its retention.

Two candidate solutions $X_{a}$ and $X_{b}$ are randomly selected from the current population. The one with the superior non-dominated rank wins; if both belong to the same rank, the individual with the larger crowding distance is retained. This process is repeated to construct the next-generation population.

From two parent individuals, a contiguous segment of the switch mapping is randomly selected and exchanged to perform the crossover operation. For example, if the controller mapping scheme is

(25) $$X_{1}=\left\{c_{1},c_{2},\ldots ,c_{s},\ldots ,c_{e},\ldots ,c_{n}\right\},\;X_{2}=\left\{c_{1}^{\prime },c_{2}^{\prime },\ldots ,c_{s}^{\prime },\ldots ,c_{e}^{\prime },\ldots ,c_{n}^{\prime }\right\}$$

then the offspring generated by crossover is

(26) $$\mathrm{swap}(X_{1}\left[s:e\right],X_{2}\left[s:e\right])\;$$

On the offspring generated by crossover, a mutation operation is performed by randomly reassigning a switch to another controller with probability $P_{m}$, introducing slight perturbations to enhance population diversity. The parent and offspring populations are then merged, and the combined set is re-sorted based on non-dominated rank and crowding distance. The top *N* individuals are selected to form the new population for the next generation. After all of the iterations are completed, the final solution is selected from the retained individuals. The overall process of the algorithm is shown in Algorithm 1.
**Algorithm 1** Dynamic Non-dominated Sorting Genetic Algorithm II (DNSGA-II)   **Input:** Time-varying graph G=(V,E,T,ϕ), controller node set $C^{\prime }$, delay matrix *D*, population size *N*, maximum number of iterations Iter, $t_{k-1}$ Time Slot Scheme *P*   **Output:** The comprehensive results of network delay $D_{\mathrm{total}}\left(t_{k}\right)$, load variance $\Delta L_{\mathrm{total}}\left(t_{k}\right)$, and normalized migration ratio $M(t_{k})$  1:Initialize the population size. Retain the optimal solution from the previous time slot and randomly generate the remaining individuals to form the initial population.  2:Evaluate the objective function values (average delay, load variance, normalized migration ratio) for all individuals in the initial population.  3:**for** i=1toIter **do**  4:       Evaluate the objective function values for all candidate solutions in the population.  5:       Perform fast non-dominated sorting to partition the population into multiple fronts.  6:       Compute the crowding distance of individuals within each front.  7:       Generate the next-generation population using tournament selection.  8:       Perform crossover and mutation operations to generate a new offspring population.  9:       Merge the parent and offspring populations.10:       Perform non-dominated sorting and crowding distance calculation again, and select the top *N* individuals with larger crowding distance to form the new population.11:**end for**

In the dynamic population initialization phase (Lines 1–3), the algorithm randomly generates initial individuals within the feasible solution space and explicitly retains the historical optimal solution from the previous time slot to complete the initial fitness evaluation, after which the main evolutionary loop begins. In Lines 4–6, the objective function values of all the candidate solutions in the population are evaluated, fast non-dominated sorting is performed to partition the population into multiple fronts, and the crowding distance of each individual is computed. In the genetic evolution phase (Lines 7–8), the algorithm selects parents using tournament selection and performs crossover and mutation operations to generate a new offspring population, thereby continuously expanding the explored solution space. In the multi-objective evaluation and environmental selection phase (Lines 9–10), the algorithm merges the parent and offspring populations, re-evaluates all solutions through non-dominated sorting and crowding distance calculation to perform multi-objective ranking, and strictly follows the criterion of “priority by rank level, and among same ranks priority by larger crowding distance” to select the top *N* elite individuals, forming the new-generation population.

### 4.2. IHAOAVOA-Based Switch Migration Mechanism

In highly dynamic satellite networks, failure recovery is often accompanied by large-scale path changes, resulting in a complex search space prone to local optima entrapment. To address this, we introduce the IHAOAVOA algorithm [24], which integrates the strengths of the Aquila Optimizer (AO) and the African Vultures Optimization Algorithm (AVOA). Specifically, it adopts the global exploration strategy of the AO and the diverse exploitation strategies of the AVOA [25], and further incorporates composite opposition-based learning (COBL) [26] and the fitness–distance balance (FDB) mechanism [27] to enhance population diversity and exploitation capability. The algorithm proceeds as follows:

First, the number of search agents *N* and the maximum number of iterations Iter are initialized, and the position of each search agent $X_{i}$ is randomly initialized. Upon entering the main iterative loop, in each iteration, boundary checking and correction are first performed for all individuals. Then, the fitness function $f(X_{i})$ is evaluated, and the current global best individual $X_{B}$ is identified. Next, the COBL strategy is employed to generate an opposition-based solution $X_{COBL}$. If the fitness of $X_{COBL}$ is superior to that of the current solution $f(X_{i})$, $X_{i}$ is replaced by $X_{COBL}$. Subsequently, the algorithm determines the current phase based on the value of the “vulture hunger coefficient” *F*. The hunger coefficient *F* is determined by the current iteration number *i* and the total number of iterations Iter, and is computed as follows:

(27) $$F=\left(2\;\cdot \;\mathrm{rand}+1\right)\;\cdot \;z\;\cdot \;\left(1-\frac{i}{Iter}\right)+g$$

where *g* is a periodic auxiliary term expressed as

(28) $$g=h\times \left(\sin\left(w\;\cdot \;\frac{\pi }{2}\;\cdot \;\frac{i}{\mathrm{Iter}}\right)+\cos\left(w\;\cdot \;\frac{\pi }{2}\;\cdot \;\frac{i}{\mathrm{Iter}}\right)-1\right)$$

where *i* denotes the current iteration number, Iter is the maximum number of iterations, *z* and *h* are random modulation factors with z∈[−1,0] and h∈[−2,2], rand is a random number taking the value 0 or 1, and *w* is a constant. If |F|≥1, the algorithm enters the exploration phase. In this phase, one of two classic update strategies from the AO algorithm is randomly selected based on a random value rand to maintain the broad search capability:

If rand≤0.5, the position is updated as follows:

(29) $$X_{i}\left(t+1\right)=X_{B}\left(t\right)\times \left(1-\frac{t}{iter}\right)+X_{m}\left(t\right)-X_{B}\left(t\right)\times rand$$

This strategy achieves directional search through the collaborative guidance of the current best solution and the mean individual. Otherwise, the FDB strategy is adopted to select the most contributive individual $X_{\mathrm{FDB}}$ from the entire population for position update:

(30) $$X_{i}\left(t+1\right)=X_{B}\left(t\right)\times Levy\left(Dim\right)+X_{\mathrm{FDB}}\left(t\right)+\left(y-x\right)\times rand$$

In this formulation, the Levy flight is employed to enable large jumps and thereby expand the search scope, while the FDB individual contributes local exploitation capability.

(31) $$Levy\left(x\right)=0.01\times \frac{u\sigma }{{\left|v\right|}^{1/\beta }}$$

(32) $$\sigma ={\left(\frac{\Gamma (1+\beta )\times \sin(\pi \beta /2)}{\Gamma \left(\left(1+\beta \right)/2\right)\times \beta \times 2^{(\beta -1)/2}}\right)}^{1/\beta }$$

where u,v∼U(0,1) are two mutually independent random variables, σ is the scale factor of the normal distribution, and β denotes the controlling exponent of the Levy distribution, typically set to β=1.5. The Levy distribution possesses a pronounced heavy-tailed characteristic, meaning that large-step jumps, though occurring with small probability, are not impossible. This property facilitates the exploration of more distant regions in the solution space, helps avoid entrapment in local optima, and enhances the capability of searching for the global optimum.

y−x represents the offset between two reference points along the search trajectory. This term originates from the construction of contour trajectories in the spiral search model and is used to simulate the rotational perturbation behavior of vultures in the search space. Through this offset vector, an individual can perform perturbative movement along a specific direction based on its current position, thereby enhancing the exploratory jump capability and diversity and avoiding entrapment in local optima.

By alternately employing these two update strategies, the exploration phase can globally search for potentially superior switch-to-controller assignment solutions. If |F|<1, the algorithm enters the exploitation phase. At this stage, the prey has been located, and multiple candidate solutions engage in intense competition around the current best solution. The algorithm further divides this phase into two behavioral stages based on the value of |F|. When |F|∈[0.5,1], it indicates that the vultures are energetic and the population is in a satiated state, simulating the following two behaviors: if $rand\leq P_{2}$, the siege-and-fight behavior among vultures is simulated. This behavioral model is given by

(33) $$X_{i}\left(t+1\right)=Dis_{i}\left(t\right)\times \left(F+rand\right)-dis_{i}\left(t\right)$$

Here, $Dis_{i}\left(t\right)$ is the directional adjustment term, and $dis_{i}\left(t\right)$ is the distance term, which together simulate the strategy where weaker vultures besiege stronger ones to seize resources. Otherwise, the circling flight behavior around the prey is simulated, with the position update formulated as

(34) $$X_{i}\left(t+1\right)=X_{B}\left(t\right)-\left(S_{1}\left(t\right)+S_{2}\left(t\right)\right)$$

where $S_{1}\left(t\right)$ and $S_{2}\left(t\right)$ are two spiral components representing the cosine and sine directional components of the spiral trajectory, respectively, and are defined as follows:

(35) $$S_{1}\left(t\right)=X_{B}\left(t\right)\times \left(\frac{rand\times X_{i}\left(t\right)}{2\pi }\right)\times \cos\left(X_{i}\left(t\right)\right).$$

(36) $$S_{2}\left(t\right)=X_{B}\left(t\right)\times \left(\frac{rand\times X_{i}\left(t\right)}{2\pi }\right)\times \sin\left(X_{i}\left(t\right)\right)$$

This behavior is used to simulate the spiral approach of vultures toward their prey. When |F|<0.5, the population enters a state of extreme hunger, triggering fierce competition for resources. At this stage:

If $rand\leq P_{3}$, the algorithm simulates all individuals launching an attack around the two best vultures, with the position update formulated as:

(37) $$X_{i}\left(t+1\right)=\frac{V_{1}\left(t\right)+V_{2}\left(t\right)}{2}.$$

where $V_{1}\left(t\right)$ and $V_{2}\left(t\right)$ are guiding components, representing the attack directions guided by the two current best vultures, respectively.

(38) $$V_{1}\left(t\right)=\frac{{\mathrm{Bestvulture}}_{1}\left(t\right)\times X_{i}\left(t\right)}{{\mathrm{Bestvulture}}_{1}\left(t\right)-X_{i}{\left(t\right)}^{2}}\times F$$

(39) $$V_{2}\left(t\right)=\frac{{\mathrm{Bestvulture}}_{2}\left(t\right)\times X_{i}\left(t\right)}{{\mathrm{Bestvulture}}_{2}\left(t\right)-X_{i}{\left(t\right)}^{2}}\times F$$

Otherwise, the remaining individuals are simulated to launch a disordered assault toward the global best individual, with the update formula as follows:

(40) $$X_{i}\left(t+1\right)=X_{B}\left(t\right)-\left|d_{i}\left(t\right)\right|\times F\times Levy\left(D\right)$$

where Levy(D) is the Levy flight operator, whose mathematical expression has been described above. $|d_{i}\left(t\right)|$ represents the Euclidean distance between the current individual $X_{i}$ and the global best solution $X_{B}\left(t\right)$, reflecting the relative positional relationship between the individual and the target and controlling the effective magnitude of the Levy jump step.

Upon completion of each iteration, the algorithm updates the counter t=t+1 until the maximum number of iterations Iter is reached. Finally, the best individual found over the entire search history is returned as the final solution of the algorithm. The overall process of the algorithm is shown in Algorithm 2.

In the initialization phase (Lines 1–2), the algorithm sets the population size and the maximum number of iterations, and randomly initializes the position of each individual. Upon entering the main loop (Lines 3–6), the individual positions are first adjusted for legality, fitness values are computed, and the two elite solutions with the best fitness ($Bestvulture_{1}$ and $Bestvulture_{2}$) are strictly selected. In the discrete opposition-based learning phase (Lines 7–12), the algorithm iterates over each agent, updates the dynamic hunger parameter *F*, performs COBL to generate a candidate solution, and applies a greedy mechanism to selectively replace the current solution, thereby enhancing the capability to escape local optima. In the global exploration phase (Lines 13–16), when the hunger coefficient |F|≥1, the algorithm alternately updates individual positions by combining the AO strategy with the FDB-based Levy flight strategy, thereby broadening the search scope of the solution space. In the local exploitation phase (Lines 17–31), when |F|<1, the algorithm further refines the exploitation behavior according to specific thresholds of |F|, performing fine-grained and in-depth mining around the current best solution. Finally, in Lines 32–35, after completing the traversal of the population, the iteration counter is updated, and the loop terminates once the maximum number of iterations is reached, at which point the optimal comprehensive switch migration solution is formally output.

### 4.3. Computational Complexity Analysis

Let $N_{p}$ denote the population size, *G* the maximum number of iterations, $N_{s}$ the number of switches to be assigned, and $N_{c}$ the number of currently available controllers. Evaluating the control-path delay, controller load variance, and normalized migration ratio of a single candidate solution requires traversing all switch-assignment variables and therefore has a time complexity of $O(N_{s})$.

For DNSGA-II, evaluating all candidate solutions in one generation requires $O(N_{p}N_{s})$ operations. Because the number of objectives is fixed at three, the worst-case complexity of fast non-dominated sorting is $O(N_{p}^{2})$. The crowding-distance calculation requires $O(N_{p}\log N_{p})$ operations, while the selection, crossover, and mutation operations require at most $O(N_{p}N_{s})$. Therefore, the overall time complexity of DNSGA-II is

$$O\;\left(G\left(N_{p}^{2}+N_{p}N_{s}\right)\right).$$

The population representation requires $O(N_{p}N_{s})$ space, while the dominance-relation lists used during non-dominated sorting may require $O(N_{p}^{2})$ space in the worst case. Hence, the overall space complexity is

$$O\;\left(N_{p}N_{s}+N_{p}^{2}\right).$$

**Algorithm 2** Improved Hybrid Aquila Optimizer–African Vultures Optimization Algorithm (IHAOAVOA)   **Input:** Time-varying graph G=(V,E,T,ϕ), controller node set $C^{\prime }$, delay matrix *D*, population size *N*, maximum number of iterations Iter.   **Output:** Comprehensive results including network delay $D_{\mathrm{total}}\left(t_{k}\right)$, load variance $\Delta L_{\mathrm{total}}\left(t_{k}\right)$, and normalized migration ratio $M(t_{k})$, returned as the final Solution  1:Initialize population size *N* and the number of iterations Iter.  2:Initialize the position of each individual.  3:**while** i≤Iter **do**  4:      Adjust positions to ensure the legality of all switch assignment decisions.  5:      Compute the fitness value of each individual in the population (integrating delay, load balancing, and normalized migration ratio).  6:      Select the two solutions with the best fitness as $Bestvulture_{1}$ and $Bestvulture_{2}$.  7:      **for** Each agent $X_{i}$ **do**  8:            Select the optimal individual $X_{B}$ via roulette wheel selection.  9:            Update the dynamic parameter *F*.10:            Perform Composite Opposition-Based Learning to generate an opposition-based candidate solution $X_{\mathrm{COBL}}$11:            **if** $f\left(X_{\mathrm{COBL}}\right)<f\left(X_{i}\right)$ **then**12:                  $X_{i}=X_{\mathrm{COBL}},\;f\left(X_{i}\right)=f\left(X_{\mathrm{COBL}}\right)$13:            **end if**14:            **if** |F|≥1 **then** Enter the Exploration phase15:                  **if** rand≤0.5 **then**16:                       update the individual using the AO strategy based on the population mean.17:                  **else**18:                       select the candidate solution farthest from the population using the FDB strategy and update the individual via Levy flight.19:                  **end if**20:            **else if** |F|<1 **then** Enter the AVOA exploitation phase21:                  **if** |F|≥0.5 **then**22:                       **if** $rand\leq P_{2}$ **then**23:                            Update the individual based on Equation.24:                       **else**25:                            Update the individual based on Equation.26:                       **end if**27:                  **else**28:                       **if** $rand\leq P_{3}$ **then**29:                            Update the individual based on Equation.30:                       **else**31:                            Update the individual based on Equation.32:                       **end if**33:                  **end if**34:            **end if**35:      **end for**36:      t←t+137:**end while**

For IHAOAVOA, the fitness evaluation, composite opposition-based learning (COBL), and discrete position-update operations require traversing the switch-assignment variables of each candidate solution. In the current implementation, population-wide statistics used by the mean-based update and the fitness–distance balance (FDB) mechanism may be recomputed for each search agent, resulting in a worst-case complexity of $O(N_{p}^{2}N_{s})$ per iteration. Moreover, selecting the preferred controllers for each switch may require sorting the $N_{c}$ available controllers, which introduces an additional complexity of $O(N_{p}N_{s}N_{c}\log N_{c})$ per iteration. Therefore, the overall worst-case time complexity of IHAOAVOA is

$$O\;\left(G\left(N_{p}^{2}N_{s}+N_{p}N_{s}N_{c}\log N_{c}\right)\right).$$

The population and the associated temporary candidate solutions require $O(N_{p}N_{s})$ space. Thus, the overall space complexity of IHAOAVOA is $O(N_{p}N_{s})$.

## 5. Simulation Experiments and Results Analysis

To validate the applicability of the two proposed switch migration mechanisms under various failure scenarios, this section constructs an SDSN dynamic simulation platform based on real orbital data and compares the performance of six representative algorithms under three failure types: link failure, controller node failure, and switch node failure. The simulations were conducted using Python 3.10 and Ansys Systems Tool Kit (STK), version 12.5, to generate time-varying topologies. The satellite constellation adopts the 66-satellite Iridium structure, and inter-satellite link data are obtained from STK dynamic snapshots. All population-based algorithms are executed with the same population size and maximum number of iterations, whereas the non-population baselines are directly applied to the same network snapshots. All methods are evaluated using the same definitions of average control-path delay, maximum control-path delay, controller load variance, and normalized migration ratio. The constellation and network configuration are listed in Table 1. The parameter settings for DNSGA-II and IHAOAVOA are listed in Table 2 and Table 3, respectively.

The six compared methods consist of four baseline methods and the two proposed methods. K-means is included as a conventional static clustering baseline. GPA assigns each switch to the controller with the minimum control-path delay and therefore represents a delay-oriented greedy baseline. NSGA-II is selected as a standard multi-objective evolutionary baseline, allowing the effect of the dynamic solution-inheritance mechanism introduced in DNSGA-II to be examined. AVOA is used as the original population-based metaheuristic baseline, allowing the effects of the hybrid AO strategy, composite opposition-based learning, and fitness–distance balance mechanisms introduced in IHAOAVOA to be evaluated. DNSGA-II and IHAOAVOA are the two proposed methods. Together, these methods cover static clustering, greedy assignment, conventional multi-objective evolutionary optimization, and population-based metaheuristic optimization.

To ensure a fair comparison, NSGA-II, AVOA, DNSGA-II, and IHAOAVOA use the same population size of 30 and the same maximum number of 50 iterations, thereby imposing a common basic search budget on all population-based algorithms. K-means and GPA do not employ population-based iterative searches and are therefore directly applied to the same network snapshots. DNSGA-II adopts a tournament size of 2, single-point crossover, and a mutation probability of 0.1. For IHAOAVOA, the parameters are set to γ=2.5, α=β=0.5, $P_{2}=0.4$, $P_{3}=0.6$, and the COBL scaling parameter k=12,000. These algorithm-specific parameters remain fixed across the link-failure, controller-node-failure, and switch-node-failure scenarios, and no failure-specific parameter retuning is performed. All methods use the same network topology, delay matrix, traffic-load vector, controller configuration, and failure state, and their output assignments are evaluated using the same metric definitions.

The population size and iteration limit are selected as a common computational budget for the population-based algorithms, rather than being adjusted to favor a particular method. For DNSGA-II, tournament selection with a size of 2 provides moderate selection pressure, while single-point crossover and a mutation probability of 0.1 preserve solution variation without excessively disrupting the switch-to-controller mappings. For IHAOAVOA, α=β=0.5 avoids an initial preference between the two reference solutions, whereas $P_{2}=0.4$ and $P_{3}=0.6$ maintain a moderate balance between the alternative exploitation behaviors. The parameter γ=2.5 controls the nonlinear transition of the search process, and k=12,000 controls the scale of the COBL operation. All parameter values are kept unchanged throughout the three failure scenarios.

The evaluation metrics include the average control-path delay, maximum control-path delay, controller load variance, and normalized migration ratio. The delay and traffic-load values are obtained from preprocessed normalized matrices. The normalized migration ratio is calculated as the number of non-controller nodes whose controller associations change, divided by the total number of network nodes. All methods are evaluated using the same representative STK-generated topology snapshots and fixed failure configurations.

To further evaluate the stochastic robustness and convergence behavior of the two proposed algorithms, DNSGA-II and IHAOAVOA were additionally tested under a representative eight-controller configuration using the initial STK topology snapshot at 04:00:00. The controller nodes in this configuration were {2,13,17,24,42,52,55,63}. To avoid selecting failure instances according to the optimization results, the failure objects were determined before the stochastic optimization process. Specifically, switch node 6 was selected as the failed switch because it had the highest traffic load among the non-controller nodes. Link (6,42) was selected as the failed link because it was the most frequently used link among the baseline switch-to-controller control paths. Controller node 42 was selected as the failed controller because it carried the largest aggregate traffic load in the baseline assignment.

After each failure was applied, the weighted shortest-path delays were recomputed using the post-failure network topology. Failed switch or controller nodes were excluded from the set of active decision variables. In the controller-failure scenario, the failed controller was also removed from the candidate controller set, thereby preventing the optimization algorithms from assigning switches to an unavailable controller. Both algorithms were evaluated using the same post-failure topology, delay matrix, traffic-load vector, controller configuration, and initial switch-to-controller assignment.

For the independent-run experiments, DNSGA-II and IHAOAVOA were each executed using 10 random seeds, namely {11,23,37,41,53,67,79,83,97,109}. The population size was fixed at 30 and the maximum number of iterations was fixed at 50 for both algorithms. The average control-path delay, controller load variance, normalized migration ratio, and runtime were recorded independently for every run. The mean, sample standard deviation, and 95% confidence interval were then calculated from the independent-run results.

Because DNSGA-II directly optimizes a set of non-dominated solutions, whereas IHAOAVOA uses a scalar fitness function, a common reporting indicator was introduced only for convergence visualization and paired statistical comparison:

(41) $$J=2D_{\mathrm{avg}}+\Delta L_{\mathrm{total}}+M$$

where $D_{\mathrm{avg}}$ denotes the normalized average control-path delay, $\Delta L_{\mathrm{total}}$ denotes the controller load variance, and *M* denotes the normalized migration ratio. A lower value of *J* indicates a better overall recovery result. This reporting indicator does not replace the original multi-objective formulation; the three original performance measures are still reported and analyzed separately.

For the statistical comparison, DNSGA-II and IHAOAVOA were paired according to their random seeds, and a two-sided exact paired randomization test was performed at a significance level of 0.05. The paired effect size was also calculated to quantify the magnitude of the difference between the two algorithms.

The main-parameter sensitivity analysis was conducted under the representative controller-failure scenario using five paired random seeds, namely {11,23,37,41,53}. A one-factor-at-a-time strategy was adopted. The population size was varied over {20,30,40} while the maximum number of iterations was fixed at 50. Subsequently, the maximum number of iterations was varied over {30,50,70} while the population size was fixed at 30. All other algorithm-specific parameters remained unchanged.

### 5.1. Experimental Results Under Link Failure Scenarios

**Figure 1:**
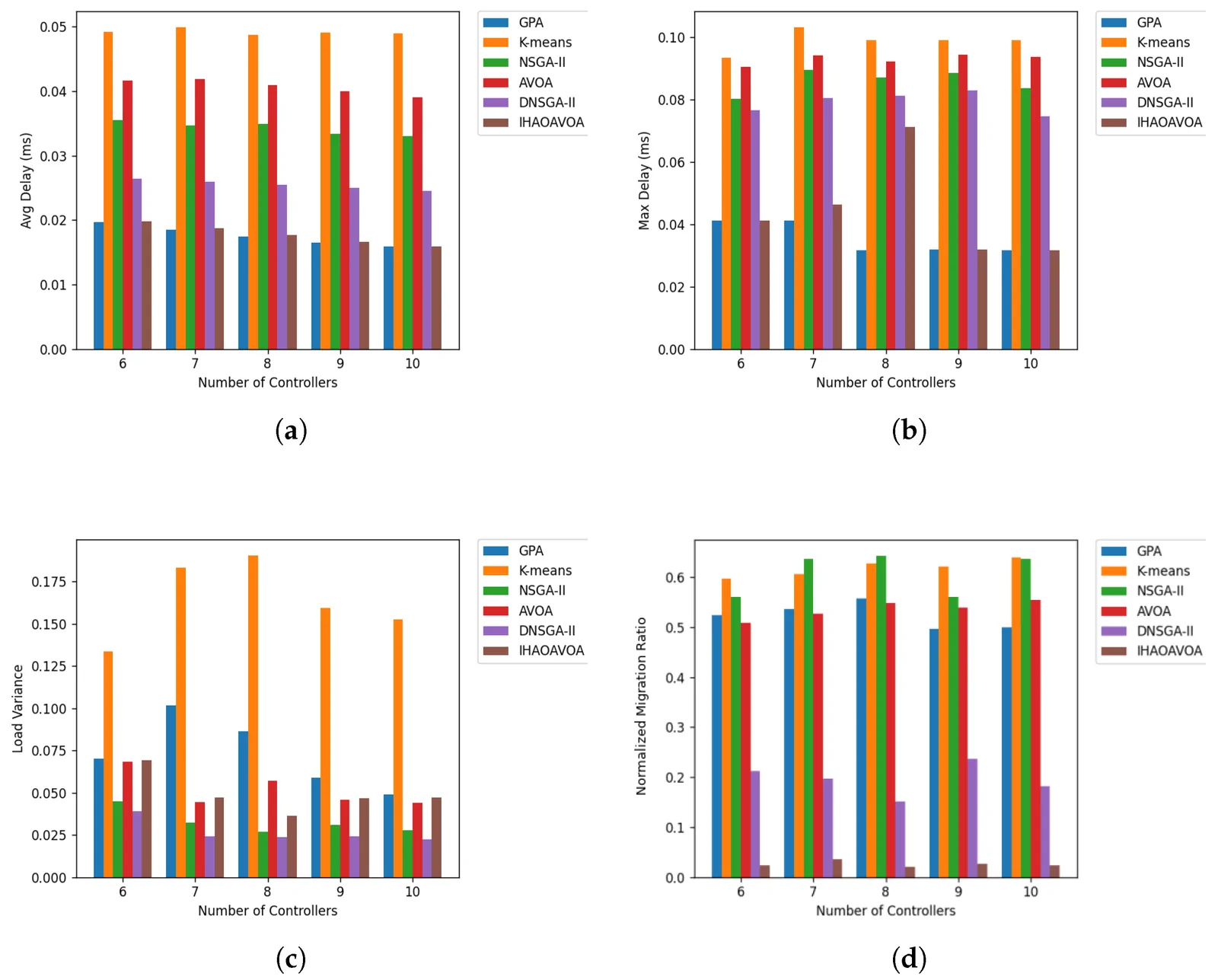
Performance comparison of K-means, GPA, NSGA-II, AVOA, DNSGA-II, and IHAOAVOA under the link-failure scenario: Lower values represent better performance for all four metrics. (**a**) Average control-path delay under the link-failure scenario. (**b**) Maximum control-path delay under the link-failure scenario. (**c**) Controller load variance under the link-failure scenario. (**d**) Normalized migration ratio under the link-failure scenario. Lower values represent better performance for all four metrics.

In the link failure scenario, certain inter-satellite links are temporarily disconnected, rendering the original control paths unavailable or degraded. The recovery capabilities of each algorithm are shown in Figure 1. Figure 1a presents the average control-path delay, Figure 1b presents the maximum control-path delay, Figure 1c presents the controller load variance, and Figure 1d presents the normalized migration ratio.

From the results, under the link failure scenario, the traditional clustering algorithm K-means, which relies solely on static distance-based partitioning, struggles to adapt to time-varying topologies and multi-objective constraints. Meanwhile, conventional metaheuristic algorithms such as NSGA-II and AVOA often lack the inheritance of historical states and are prone to falling into local optima when facing abrupt network state changes, leading to suboptimal overall performance. As shown in Figure 1a, IHAOAVOA achieves the lowest average delay among the compared methods, achieving a reduction of over 24–63% compared to K-means, NSGA-II, AVOA, and DNSGA-II, respectively. This is largely attributable to the composite opposition-based learning and broad exploration strategy it incorporates, which effectively expand the solution space and enable it to rapidly escape local traps after link damage, thereby constructing high-quality control paths. Meanwhile, Figure 1b shows that IHAOAVOA achieves the lowest maximum control-path delay among the compared methods in the evaluated link-failure scenario, indicating favorable recovery performance under severe path degradation.

From Figure 1b,c, it can be observed that DNSGA-II exhibits a slight disadvantage in terms of maximum delay, yet achieves the lowest load variance among the compared methods, with lower load fluctuation than IHAOAVOA and the conventional algorithms in the evaluated scenario. This is because DNSGA-II does not solely pursue extremely low delay; rather, it rigorously mitigates the conflicting imbalance among multiple objectives through the non-dominated sorting and crowding distance mechanism, indicating that DNSGA-II is more prominent in maintaining resource equilibrium and avoiding secondary overload of local controllers.

In terms of normalized migration ratio, as shown in Figure 1d, conventional algorithms tend to perform global reassignment after a failure, leading to drastic fluctuations in switch-to-controller associations. In contrast, IHAOAVOA achieves a reduction in normalized migration ratio of 75–96% relative to the compared baselines, resulting in the lowest normalized migration ratio among the compared methods in this scenario. This is attributed to its multi-state dynamic fine-tuning mechanism, which performs adaptive remapping only on affected nodes when local network fluctuations occur, thereby minimizing unnecessary handovers. Meanwhile, Figure 1d also indicates that DNSGA-II benefits from inheriting the optimal solution from the previous time slot during initialization, effectively avoiding blind network-wide reconfiguration. Although it exhibits a slightly higher normalized migration ratio than IHAOAVOA, its normalized migration ratio remains lower than that of most conventional algorithms in this scenario.

### 5.2. Experimental Results Under Controller Node Failure Scenarios

**Figure 2:**
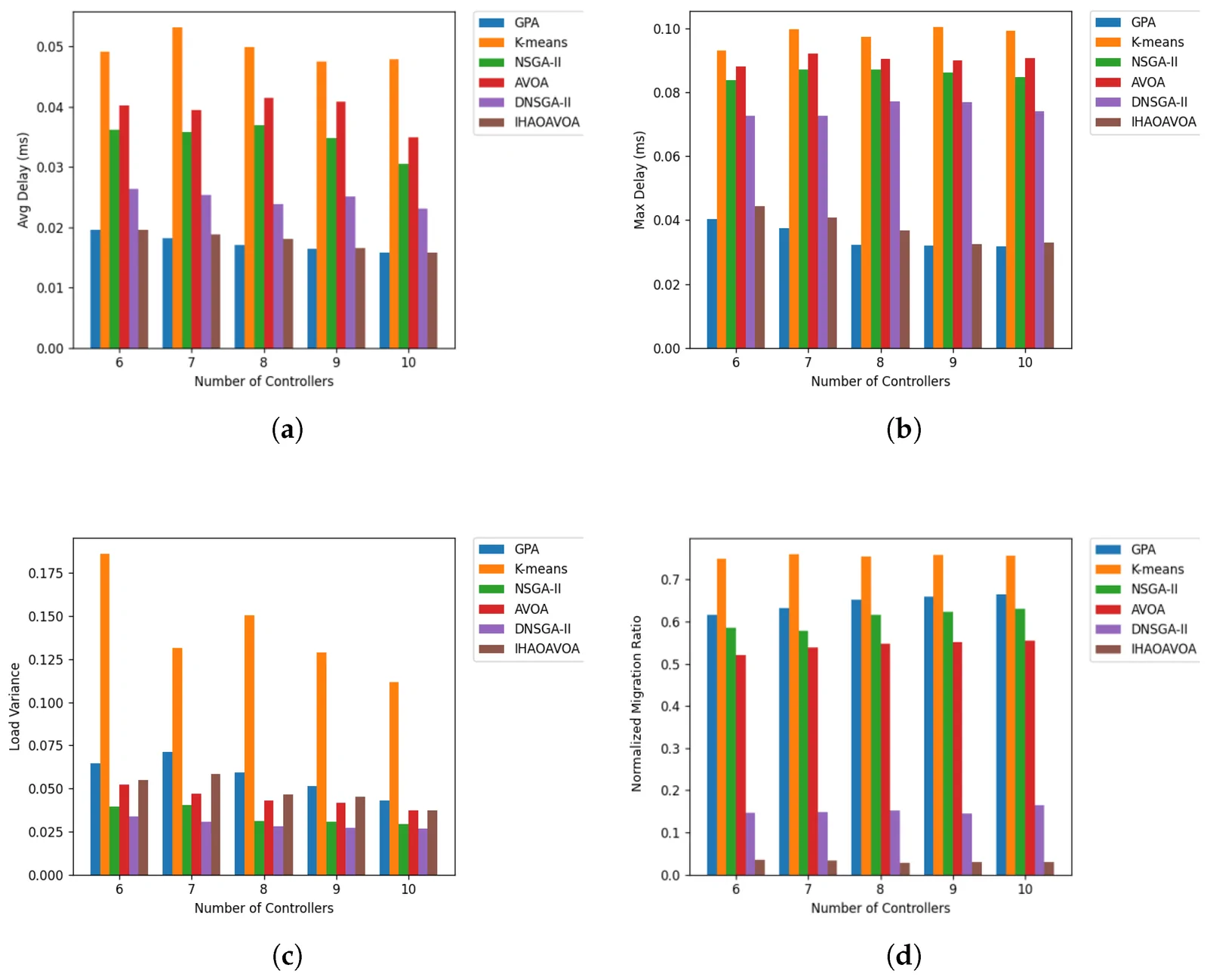
Performance comparison of K-means, GPA, NSGA-II, AVOA, DNSGA-II, and IHAOAVOA under the controller-node-failure scenario: (**a**) Average control-path delay under the controller-node-failure scenario. (**b**) Maximum control-path delay under the controller-node-failure scenario. (**c**) Controller load variance under the controller-node-failure scenario. (**d**) Normalized migration ratio under the controller-node-failure scenario. Lower values represent better performance for all four metrics.

When a controller node fails, a subset of switches across the entire network must be reassigned to new controllers, making this scenario more demanding in terms of testing the stability and complex-environment handling capability of the migration strategies. The results are shown in Figure 2, where Figure 2a presents the average control-path delay, Figure 2b presents the maximum control-path delay, Figure 2c presents the controller load variance, and Figure 2d presents the normalized migration ratio.

In this scenario, the failure of a controller node causes a large number of switches within its domain to instantaneously lose their control links. When confronted with such abrupt topological changes, conventional static algorithms and standard metaheuristic algorithms, lacking an efficient dynamic optimization mechanism, often struggle to promptly assign appropriate alternative controllers to the affected nodes. As shown in Figure 2a, the average delay of IHAOAVOA is comparable to that of GPA, yet it achieves reductions of nearly 60%, 44%, and 52% compared to K-means, NSGA-II, and AVOA, respectively. This indicates that IHAOAVOA, by leveraging global guidance and large-span search strategies, can effectively escape local optima and rapidly reconstruct low-latency control paths for disconnected switches after a severe failure. Figure 2b shows a similar trend in terms of maximum delay, where IHAOAVOA achieves the lowest maximum control-path delay among the compared methods, with a reduction of approximately 39% in the evaluated controller-node-failure scenario.

As can be observed from Figure 2a,b, DNSGA-II exhibits inferior average and maximum delays compared to IHAOAVOA in this scenario, with an increase of 25–33%. Nevertheless, as shown in Figure 2c, DNSGA-II achieves the lowest load variance among the compared methods in this scenario. The main reason is that the concentrated traffic shift caused by a single controller failure can readily induce cascading overload in other healthy controllers. DNSGA-II, by virtue of its multi-objective ranking mechanism, imposes strict constraints on load variance, thereby trading off a certain degree of delay performance in exchange for the stable distribution of control resources across the entire network.

In terms of normalized migration ratio, as shown in Figure 2d, IHAOAVOA achieves a reduction generally exceeding 93%. This is primarily because its local adaptive fine-tuning mechanism effectively avoids unnecessary remapping of nodes in unaffected regions while restoring the failed area. In contrast, as can be seen from Figure 2d, DNSGA-II exhibits a higher normalized migration ratio under controller failure, even surpassing that of some conventional algorithms. This is mainly because DNSGA-II, in pursuit of global load balancing, needs to disrupt the original assignment states of normal regions, triggering large-scale node reassignment. This also objectively reflects that, when facing core node failures, DNSGA-II must incur a high network reconfiguration cost to maintain stable system load.

### 5.3. Experimental Results Under Switch Node Failure Scenarios

**Figure 3:**
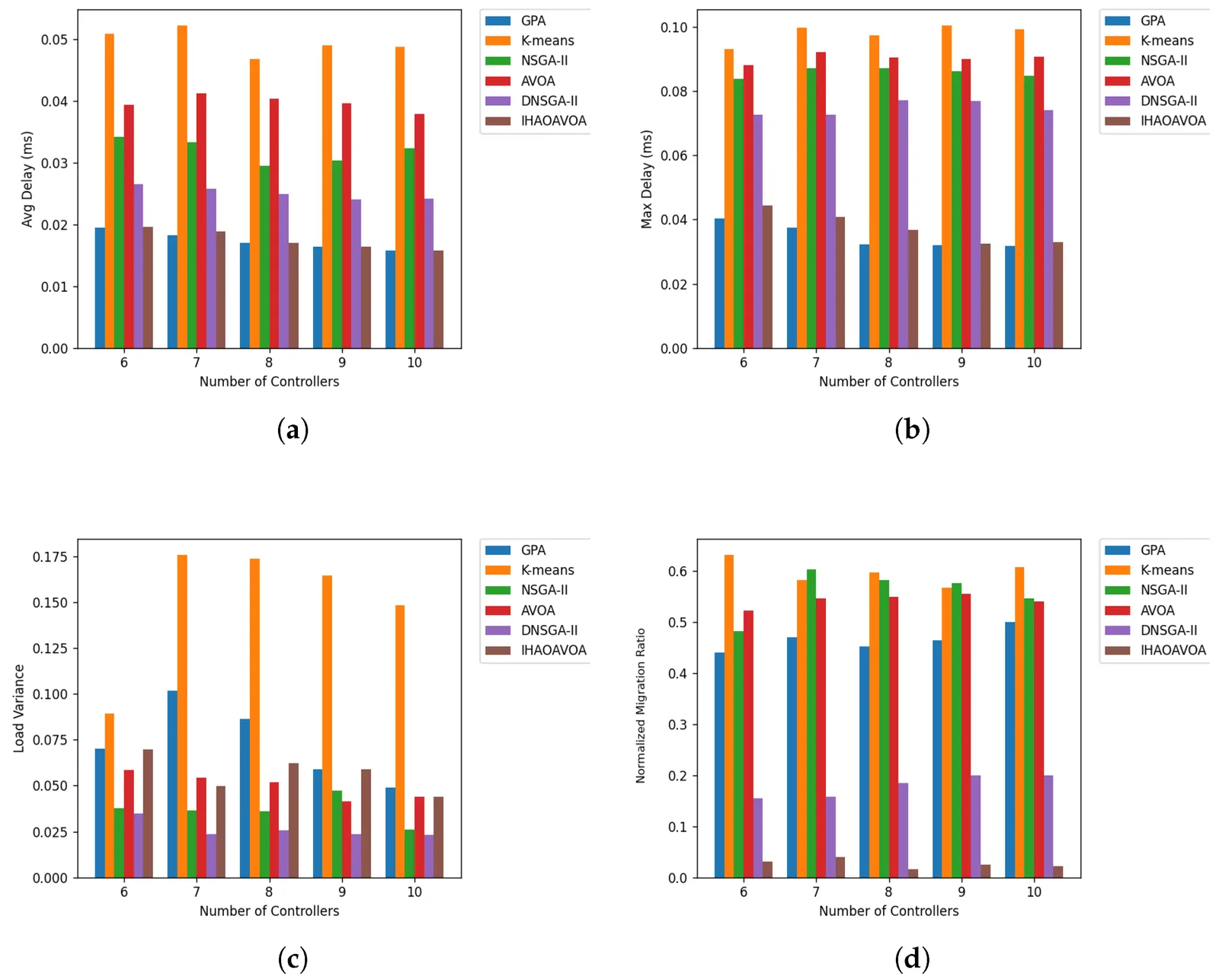
Performance comparison of K-means, GPA, NSGA-II, AVOA, DNSGA-II, and IHAOAVOA under the switch-node-failure scenario: (**a**) Average control-path delay under the switch-node-failure scenario. (**b**) Maximum control-path delay under the switch-node-failure scenario. (**c**) Controller load variance under the switch-node-failure scenario. (**d**) Normalized migration ratio under the switch-node-failure scenario. Lower values represent better performance for all four metrics.

Switch node failures result in the complete disconnection of some data plane nodes from the control plane. Such failures are relatively common in spaceborne networks, particularly when energy is insufficient or attitude is constrained. Figure 3 illustrates the recovery performance of the compared algorithms under this scenario, where Figure 3a presents the average control-path delay, Figure 3b presents the maximum control-path delay, Figure 3c presents the controller load variance, and Figure 3d presents the normalized migration ratio.

In the scenario of switch node failure, the damage to the local topology triggers the passive transfer of surrounding traffic. Conventional static clustering and standard heuristic algorithms, when confronted with such local disturbances, often lack the precision for fine-tuning and are prone to triggering unnecessary large-scale remapping. As can be seen from Figure 3a,b, IHAOAVOA still demonstrates the most stable delay performance, with both its average delay and maximum delay maintained at low levels without drastic fluctuations caused by switch loss. This is largely attributable to its multi-state refined search mechanism during the local exploitation phase, which enables it to rapidly lock onto optimal alternative paths within the failure neighborhood when the local topology is damaged. In contrast, DNSGA-II exhibits a relatively larger increase in average delay. However, as shown in Figure 3c, it continues to maintain its advantage in load balancing, achieving a more even distribution of tasks among controllers. The main reason is that DNSGA-II, relying on non-dominated sorting, strictly constrains the multi-objective balance during the traffic transfer process, preferring to sacrifice some directness of control paths to prevent the redistribution of local traffic from disrupting the global load balancing state of the controllers.

In terms of normalized migration ratio, as shown in Figure 3d, IHAOAVOA maintains its consistent advantage, with a lower normalized migration ratio than the other compared algorithms. Even when node losses lead to a redistribution of the mapping space, IHAOAVOA converges rapidly in the evaluated switch-node-failure scenario. This result suggests that its adaptive adjustment strategy can limit the impact of failure recovery to the vicinity of the damaged nodes.

In contrast, DNSGA-II, as can be observed from Figure 3d, still incurs a relatively high normalized migration ratio. This is because its stringent pursuit of global load balancing inevitably triggers collateral migration of numerous normal nodes. Nevertheless, benefiting from its memory inheritance of the historical optimal solution, DNSGA-II still achieves a certain optimization effect compared to the exorbitant overhead of global blind reconfiguration exhibited by traditional clustering methods.

### 5.4. Convergence, Statistical Robustness, and Parameter Sensitivity

#### 5.4.1. Convergence Behavior

**Figure 4:**
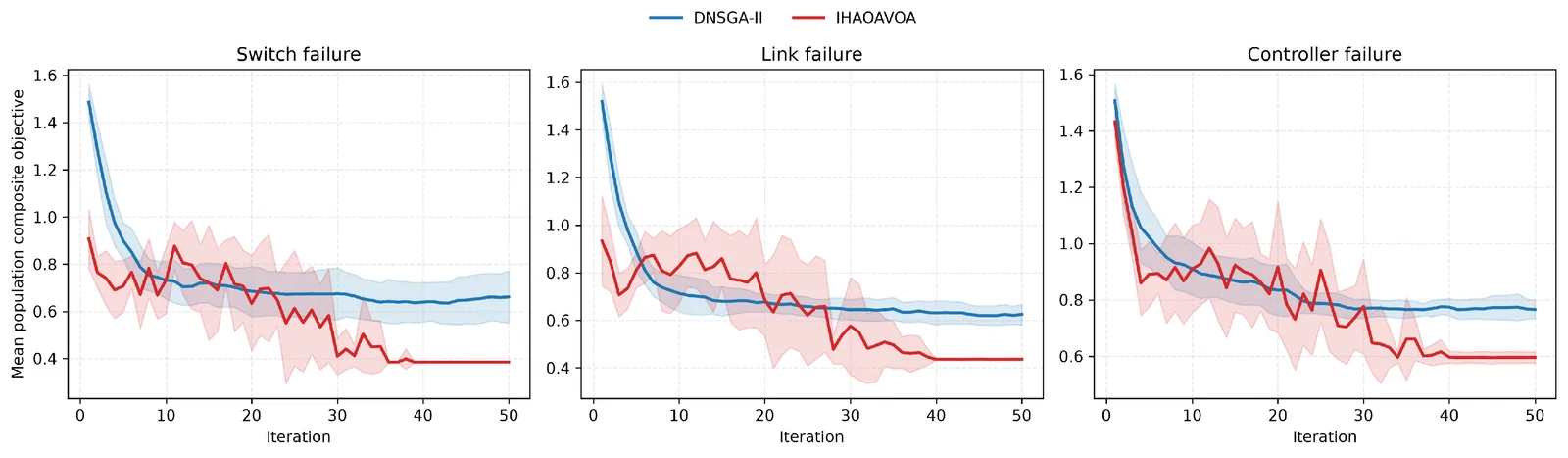
Convergence comparison of DNSGA-II and IHAOAVOA under the representative switch-failure, link-failure, and controller-failure scenarios. The solid lines show the mean population-average reporting indicator over 10 independent random seeds, and the shaded regions show ± one sample standard deviation. Lower values indicate better overall recovery performance.

Figure 4 presents the convergence behavior of DNSGA-II and IHAOAVOA under the representative switch-failure, link-failure, and controller-failure scenarios. Because DNSGA-II directly maintains a set of non-dominated solutions whereas IHAOAVOA employs a scalar fitness function, the common reporting indicator defined in Equation (41) is used to provide a consistent convergence comparison. Each curve represents the across-seed mean of the population-average reporting indicator over 10 independent runs, and the shaded region represents one sample standard deviation.

Under the switch-failure scenario, the mean population-average reporting indicator of DNSGA-II decreases from 1.4865 in the first iteration to 0.6616 in the 50th iteration, while that of IHAOAVOA decreases from 0.9070 to 0.3845. Under the link-failure scenario, the corresponding indicator decreases from 1.5189 to 0.6244 for DNSGA-II and from 0.9333 to 0.4353 for IHAOAVOA. Under the controller-failure scenario, DNSGA-II decreases from 1.5069 to 0.7668, whereas IHAOAVOA decreases from 1.4324 to 0.5962.

These results provide empirical evidence that both algorithms progressively improve their population quality within the adopted 50-iteration search budget. DNSGA-II exhibits a relatively gradual evolutionary process because its non-dominated sorting and diversity-maintenance mechanisms preserve solutions with different objective preferences. In contrast, IHAOAVOA generally reaches a lower reporting indicator and demonstrates a faster reduction during the later search stage, which is consistent with its exploitation-oriented update and greedy candidate-retention mechanisms.

#### 5.4.2. Independent-Run Statistics and Significance

Table 4 reports the mean and sample standard deviation obtained from 10 independent runs. The separate performance objectives are retained in the table because the reporting indicator *J* is introduced only to provide a common convergence and statistical comparison, rather than to replace the original multi-objective evaluation.

IHAOAVOA obtains a lower average control-path delay and normalized migration ratio in all three representative failure scenarios. DNSGA-II, however, consistently produces a lower controller load variance. This observation confirms the complementary characteristics of the two proposed algorithms. IHAOAVOA places greater emphasis on rapid delay recovery and limited switch reassignment, whereas DNSGA-II places greater emphasis on the balanced distribution of traffic among the available controllers.

To determine whether the differences in the common reporting indicator are caused only by stochastic variation, a two-sided exact paired randomization test was performed by pairing the two algorithms according to their random seeds. For the reporting indicator *J*, the test gives p=0.001953 under the switch-failure, link-failure, and controller-failure scenarios. All three values are below the significance level of 0.05, indicating that the lower mean reporting indicator obtained by IHAOAVOA is statistically significant in the evaluated representative configurations.

The paired effect sizes are 9.775, 5.518, and 7.337 under switch failure, link failure, and controller failure, respectively. These values indicate that the observed differences in the reporting indicator are large relative to the paired across-seed variation. Nevertheless, this statistical result should be interpreted together with the individual objectives in Table 4. Although IHAOAVOA achieves a lower composite reporting indicator, DNSGA-II retains a clear advantage in controller load variance under all three failure types. Therefore, the statistical analysis supports the complementary use of the two algorithms rather than the universal superiority of a single algorithm.

The zero standard deviation obtained by IHAOAVOA for several metrics under the representative switch-failure scenario indicates that all 10 independent runs returned the same final switch-to-controller assignment. This result suggests that the corresponding recovery state has a dominant solution that can be consistently identified by IHAOAVOA under the adopted search budget.

#### 5.4.3. Sensitivity to the Main Computational Parameters

The sensitivity analysis focuses on the population size and the maximum number of iterations because these two parameters directly determine the search diversity, convergence opportunity, and computational cost of both population-based algorithms. Moreover, they can be varied under an identical experimental budget for DNSGA-II and IHAOAVOA, which avoids introducing algorithm-specific advantages during the comparison. All other parameters, including the mutation probability of DNSGA-II and the adaptive search parameters of IHAOAVOA, were maintained at the values reported in Table 2 and Table 3.

A one-factor-at-a-time sensitivity analysis was conducted under the representative controller-failure scenario. Five paired random seeds, namely {11,23,37,41,53}, were used for every tested setting. When evaluating the effect of population size, the population size was varied over {20,30,40} while the maximum number of iterations was fixed at 50. When evaluating the effect of the iteration limit, the maximum number of iterations was varied over {30,50,70} while the population size was fixed at 30. The final reporting indicator *J* was recorded for every independent run.

**Figure 5:**
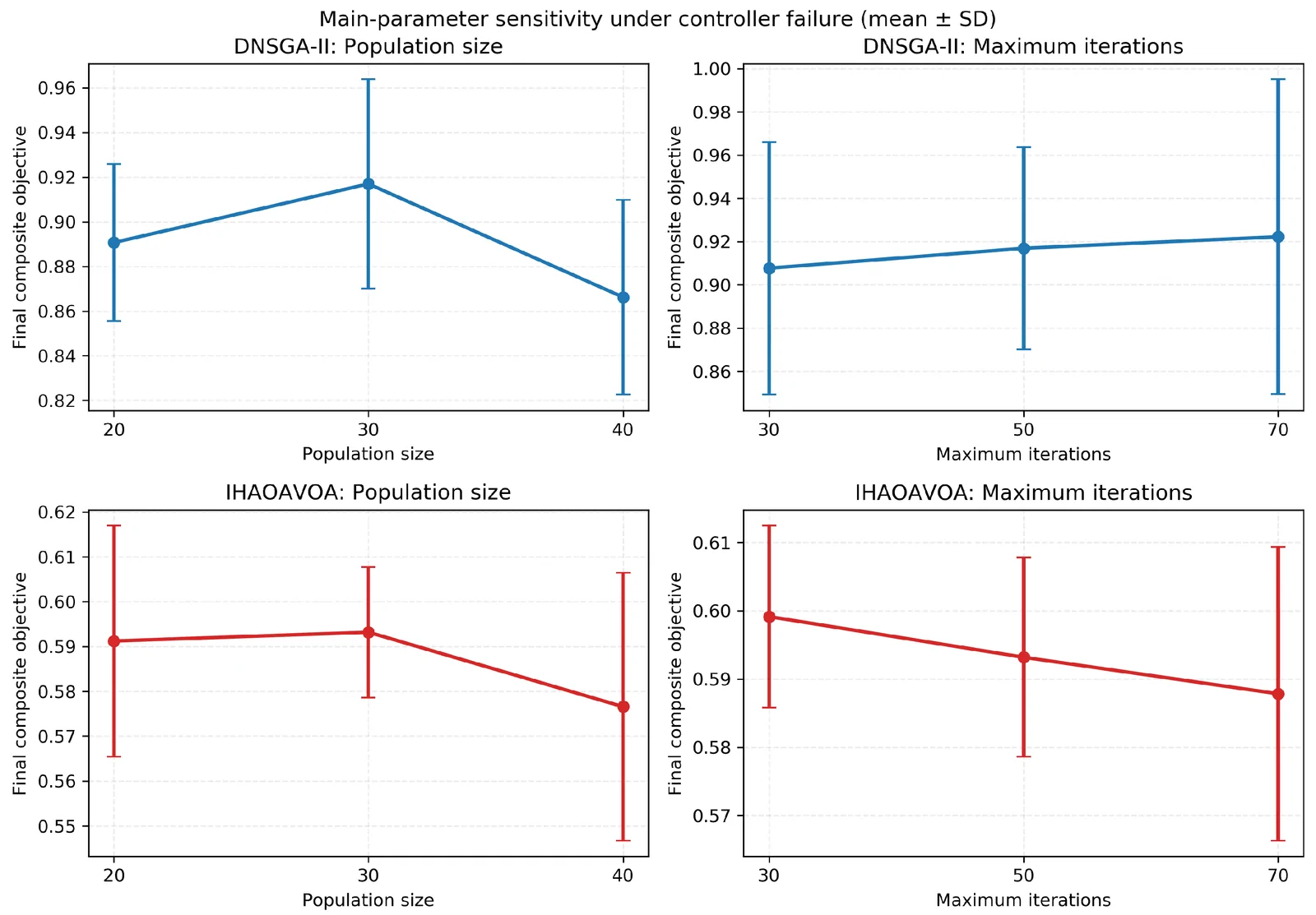
Sensitivityof DNSGA-II and IHAOAVOA to the population size and maximum number of iterations under the representative controller-failure scenario. Each point represents the mean final reporting indicator over five paired random seeds, and the error bars represent ± one sample standard deviation. Lower values indicate better overall recovery performance.

As shown in Figure 5, the performance of DNSGA-II does not change monotonically with either the population size or the maximum number of iterations. When the population size is set to 20, 30, and 40, the mean final reporting indicators are 0.890660, 0.916906, and 0.866234, respectively, with sample standard deviations of 0.035090, 0.046780, and 0.043571. A population size of 40 produces the lowest tested mean indicator; however, the improvement over the smaller population settings is limited relative to the additional fitness evaluations required by the larger population.

When the maximum number of iterations is set to 30, 50, and 70, the corresponding mean reporting indicators of DNSGA-II are 0.907668, 0.916906, and 0.922206, respectively. Their sample standard deviations are 0.058414, 0.046780, and 0.072790. Increasing the iteration limit therefore does not yield a systematic improvement for DNSGA-II. This behavior is related to its multi-objective non-dominated selection mechanism: additional generations may preserve alternative Pareto solutions that favor load balancing rather than continuously decreasing the scalar reporting indicator. Consequently, the reporting indicator should not be interpreted as the sole optimization objective of DNSGA-II.

For IHAOAVOA, the mean reporting indicators obtained with population sizes of 20, 30, and 40 are 0.591214, 0.593183, and 0.576574, respectively. The corresponding sample standard deviations are 0.025753, 0.014565, and 0.029842. The differences among the tested population sizes are relatively small, although a population size of 40 achieves the lowest tested mean value.

When the iteration limit is increased from 30 to 50 and 70, the mean reporting indicator of IHAOAVOA decreases from 0.599133 to 0.593183 and 0.587834, respectively. The corresponding sample standard deviations are 0.013348, 0.014565, and 0.021499. Thus, additional iterations provide a modest improvement for IHAOAVOA, but the magnitude of the improvement becomes limited when compared with the additional computational cost. This result indicates that IHAOAVOA is not highly sensitive to moderate changes in the adopted iteration limit.

Overall, the comparative performance trends of DNSGA-II and IHAOAVOA remain stable over the tested parameter ranges. DNSGA-II continues to emphasize controller load balancing, whereas IHAOAVOA continues to favor a lower delay and normalized migration ratio. Although some larger parameter settings produce slightly lower reporting indicators, they also require proportionally more candidate evaluations.

Therefore, a population size of 30 and a maximum number of 50 iterations are retained as a balanced common computational budget. These values provide sufficient search diversity and convergence opportunity without imposing the higher computational overhead associated with the largest tested settings. The sensitivity results do not imply that the adopted parameters are globally optimal; instead, they demonstrate that the principal conclusions of the comparison are not caused by a single narrowly selected parameter configuration.

## 6. Conclusions

This study formulated a failure-recovery-oriented switch migration model for SDSNs that jointly considers average control-path delay, controller load variance, and normalized migration ratio. To address different recovery priorities, we developed two complementary mechanisms. DNSGA-II uses historical-solution inheritance and diversity maintenance to preserve load-balanced assignments, whereas IHAOAVOA combines adaptive exploration and exploitation with discrete search mechanisms to achieve rapid recovery with limited switch reassignment.

Experiments under link-failure, controller-node-failure, and switch-node-failure scenarios showed distinct but complementary performance profiles. Across 10 independent runs, both algorithms progressively improved their population quality within the adopted 50-iteration search budget. IHAOAVOA generally achieved lower average control-path delay and normalized migration ratio, whereas DNSGA-II consistently achieved lower controller load variance. For the common reporting indicator *J*, the two-sided exact paired randomization tests yielded p=0.001953 in all three evaluated representative scenarios. Because *J* is used only for convergence visualization and paired statistical comparison, these statistical results should be interpreted together with the individual objectives. Therefore, the results support complementary algorithm selection rather than the universal superiority of a single method.

The sensitivity analysis further showed that the main comparative trends remained stable when the population size varied from 20 to 40 and the maximum number of iterations varied from 30 to 70. Accordingly, a population size of 30 and a maximum of 50 iterations were retained as a balanced common computational budget rather than as globally optimal settings. The present conclusions are limited to the evaluated Iridium-based topology snapshots, failure configurations, and normalized input data. Future work will investigate larger and more heterogeneous constellations, additional resource and reliability constraints, and cross-layer recovery mechanisms for satellite-terrestrial integrated networks.

## Figures and Tables

**Table 1:** Constellation and network configuration.

Constellation Type	Number of Satellites	Number of Orbital Planes	Orbital Inclination	Orbital Altitude
Iridium constellation	66	6	86.4°	780 km

**Table 2:** DNSGA-II algorithm parameters.

Pop. Size	Max. Iter	Selection	Crossover	Mut. Prob	Diversity Maintenance
30	50	Tournament (size = 2)	Single-point	0.1	NDS + CD

**Table 3:** IHAOAVOA algorithm parameters.

Pop. Size	Max. Iter	γ	$P_{2},P_{3}$	COBL *k*
30	50	2.5	0.4, 0.6	12,000

**Table 4:** Independent-run results under the three representative failure scenarios. Each value is reported as the mean ± sample standard deviation over 10 independent random seeds. Lower values indicate better performance.

Failure Scenario	Algorithm	Average Delay	Load Variance	Migration Ratio	Reporting Indicator *J*
Switch failure	DNSGA-II	0.269294±0.006844	0.000742±0.000193	0.237879±0.031982	0.777210±0.040533
Switch failure	IHAOAVOA	0.185696±0.000000	0.009595±0.000000	0.000000±0.000000	0.380987±0.000000
Link failure	DNSGA-II	0.265042±0.011634	0.026620±0.000348	0.269697±0.052874	0.826401±0.071746
Link failure	IHAOAVOA	0.180266±0.000802	0.053294±0.003542	0.018182±0.006388	0.432008±0.001243
Controller failure	DNSGA-II	0.274380±0.004519	0.027215±0.000496	0.330303±0.031782	0.906278±0.033467
Controller failure	IHAOAVOA	0.190708±0.003408	0.080770±0.026508	0.122727±0.004791	0.584913±0.021052

## Data Availability

The raw data supporting the conclusions of this article will be made available by the authors on request.
